# Fine Mapping and Cloning of a Major QTL *qph12*, Which Simultaneously Affects the Plant Height, Panicle Length, Spikelet Number and Yield in Rice (*Oryza sativa* L.)

**DOI:** 10.3389/fpls.2022.878558

**Published:** 2022-05-27

**Authors:** Niqing He, Guanping Zhan, Fenghuang Huang, Salah Fatouh Abou-Elwafa, Dewei Yang

**Affiliations:** ^1^Rice Research Institute, Fujian High Quality Rice Research and Development Center, Fujian Academy of Agricultural Sciences, Fuzhou, China; ^2^College of Life Sciences, Fujian Agriculture and Forestry University, Fuzhou, China; ^3^Faculty of Agriculture, Department of Agronomy, Assiut University, Assiut, Egypt

**Keywords:** rice (*Oryza sativa* L. subsp. *indica*), gene mapping, gene cloning, auxin, near-isogenic lines

## Abstract

Plant height is one of the most important agronomical traits in rice (*Oryza sativa* L.). Introducing the *semidwarf* rice in the 1960s significantly enhanced the rice yield potential in Asia. Implementing near-isogenic lines (NILs) is the most powerful tool for the identification and fine mapping of quantitative trait loci (QTLs). In this study, 176 NILs were produced from the crossing and back-crossing of two rice cultivars. Specifically, the *indica* rice cultivar Jiafuzhan served as a recipient, and the restorer *japonica* cultivar Hui1586 served as a donor. Using the 176 NILs, we identified a novel major QTL for reduced plant height in the NIL36 line. The *qph12* QTL was mapped to a 31 kb genomic region between the indel markers *Indel12-29* and *Indel12-31*. The rice genome annotation indicated the presence of three candidate genes in this genomic region. Through gene prediction and cDNA sequencing, we confirmed that *LOC_Os12g40890* (*qPH12*) is the target gene in the NIL36 line. Further analysis showed that the *qph12* QTL is caused by a 1 bp deletion in the first exon that resulted in premature termination of the *qPH12*. Knockout experiments showed that the *qph12* QTL is responsible for the reduced plant height phenotype of the NIL36 line. Although the *qph12* gene from the NIL36 line showed a shorter panicle length, fewer spikelets per panicle and a lower plant grain yield, the plant also exhibited a lower plant height. Taken together, our results revealed that the *qph12* have good specific application prospects in future rice breeding.

## Introduction

Plant height is an important factor that determines the architecture and grain yield of cereal plants (Wang et al., 2018; Liao et al., 2019). The *semidwarf* genes, which result in a shortened culm, improved lodging resistance and an increased harvest index, contributed to the “Green Revolution” in wheat and rice (Peng et al., 1999; Spielmeyer et al., 2002). However, the wide application of dwarf germplasm resources and their narrow genetic range coupled with the excessive use of pesticides and fertilizers have led to serious environmental problems (Pimentel, 1996; Deng et al., 2019), and these problems have encouraged the study of genetic and molecular mechanisms for establishing an “ideal” plant structure through regulating plant height.

Several QTLs associated with plant height have been identified in rice (Li et al., 2003; Zhang et al., 2006; Lee et al., 2014). Moreover, using RILs, Han et al. (2017) identified three QTLs designated *qPh3.1*, *qPh1* and *qPh7.1* for plant height. Furthermore, using the CSSL population, Shearman et al. (2019) identified plant height QTLs on chromosomes 1 and 4. Most of the identified genes related to plant height, such as *semidwarf1* (*sd1*; Sasaki et al., 2002), *GA-insensitive dwarf1* (*gid1*; Ueguchi-Tanaka et al., 2005), *GA-insensitive dwarf2* (*gid2*; Hirano et al., 2010), *BR-deficient dwarf1* (*brd1*; Mori et al., 2002), *BR-insensitive mutant* (*d61*; Hong et al., 2003), and *BR-deficient mutant* (*osdwarf4-1*; Sakamoto et al., 2006), are related to the metabolism or signaling of the phytohormones gibberellin (GA) and brassinosteroid (BR; Deng et al., 2019).

Although the GA- and BR-related genes associated with plant height have been extensively studied, an increasing number of novel plant height-related genes that rely on pathways other than the GA and BR pathways have been identified. For instance, the carotenoid-derived phytohormone strigolactone has become a focus of research on plant architecture patterning (Zhou et al., 2013). Recent studies have shown that *OsCKX9*, which encodes a cytokinin oxidase that catalyzes the degradation of cytokinin, functions as a primary strigolactone-responsive gene that regulates rice tillering, plant height, and panicle size, likely *via* the secondary response gene *OsRR5*, which encodes a cytokinin-inducible rice type-A response regulator. This pathway demonstrates that strigolactone regulates the rice shoots architecture by enhancing cytokinin catabolism through modulating the expression of *OsCKX9* (Duan et al., 2019).

Auxin exerts pleiotropic effects on plant cell elongation, cell division and differentiation, root initiation, apical dominance, and tropic responses by regulating the expression of the early auxin-responsive *auxin/indoleacetic acid* (*Aux/IAA*) genes (Jain et al., 2006). By screening the publicly available databases, Jain et al. (2006) identified 31 *Aux/IAA* genes in rice and found that these genes had different functions. *OsIAA1* and *OsIAA3* play important roles in the crosstalk between the auxin and brassinosteroid signaling pathways and plant morphogenesis (Thakur et al., 2001; Nakamura et al., 2006). The gain-of-function mutation in *OsIAA11* inhibits lateral root development in rice (Zhu et al., 2012). *OsIAA13*-mediated auxin signaling is involved in lateral root initiation in rice (Kitomi et al., 2012). *OsIAA6* is involved in drought tolerance and tiller outgrowth (Jung et al., 2015), and the *OsIAA10* protein directly targets the rice dwarf virus P2 protein and enhances viral infection and disease development (Jin et al., 2016). Near-isogenic lines (NILs) carried one or more donor chromosome segments provide distinct advantages for QTL identification (Yang et al., 2016). Moreover, NILs can block background genetic noise, undoubtedly enhance our understanding of complex traits and promote plant genomic studies (Henry et al., 2015; Yang et al., 2016).

The current study was carried out to: (i) develop a rice NIL population through the crossing and back-crossing of two rice cultivars, i.e., the *japonica* cultivar Hui1586 that served as a donor, and the *indica* cultivar Jiafuzhan that served as a recipient, (ii) implementing 176 NILs for fine mapping of major QTLs and cloning of genes underlying plant height using, (iii) performing a functional analysis of the cloned genes using CRISPR/Cas9 genome editing.

## Materials and Methods

### Plant Materials

The *indica* rice cultivar Jiafuzhan and the *japonica* rice cultivar Hui1586 were preserved at the Rice Research Institute, Fujian Academy of Agricultural Sciences, China. Hui1586 was derived from a cross between Suxiu867 and Minghui86, and the detailed selection process was as follows: Suxiu867 (Food Crops Research Institute, Jiangsu Academy of Agricultural Sciences), a *japonica* cultivar, was used as a recipient, and Minghui86 (Rice Research Institute, Fujian Academy of Agricultural Sciences), a restorer *indica* cultivar, was used as a donor. The F_1_ plants were generated from Suxiu867 as the female parent and Minghui86 as the male parent. The F_1_ plants were backcrossed to the Suxiu867 parent to produce the BC_1_F_1_ generation. Six BC_1_F_1_ plants were backcrossed to the Suxiu867 parent to produce 6 BC_2_F_1_ plants, which were self-pollinated to produce 72 BC_2_F_2_ lines (12 individuals from each of the six BC_2_F_1_ plants was sown). The 72 individuals were self-pollinated for six generations. In this process, shorter plants, higher seed setting rate and better comprehensive agronomic traits were selected for to continue planting single plant and eliminate the remaining lines. Then a stable line designated Hui1586 was obtained.

### NILs Development

For the development of the NILs, the *indica* cultivar Jiafuzhan was used as a recipient, and the restorer *japonica* cultivar Hui1586 was used as a donor. The F_1_ plants were generated from the Jiafuzhan as the female parent and Hui1586 as the male parent. The F_1_ plants were back-crossed to the Jiafuzhan parent to produce the BC_1_F_1_ generation. These BC_1_F_1_ plants were then backcrossed to the Jiafuzhan parent to produce BC_2_F_1_ plants. Using the same approach, 118 BC_3_F_1_ individuals were obtained, which were self-pollinated to produce the BC_3_F_2_ lines. Based on their characteristics, we selected one or two individual plants from each line. As a result, 176 NILs were obtained (Supplementary Figure 1).

### QTL Analysis

The WinQTLCart 2.5 software (Wang et al., 2012) implementing the composite interval mapping (CIM) was employed for QTL detection (Zeng, 1993). The confidence interval was defined as the 1-LOD reducing region around the locus of a peak LOD value of the identified QTL. LOD value of ≥ 2.5 was set as a threshold for QTL identification.

### Identification of Major QTLs for Plant Height

In autumn 2019, Jiafuzhan, Hui1586 and 176 NILs were planted under natural conditions in the paddy fields of Sanya Experimental Station, Hainan Province, China (18°14′N, 109°31′E, 7^.^0 m asl). Forty-eight plants of each of the parents and the NILs were planted in six rows. Four plants from the center of each plot were picked to evaluate their plant height characteristics. Major QTLs associated with plant height were identified on the basis of significant differences in plant height between parents and each of the NILs, as determined by *t*-test. Moreover, panicle length, effective panicle number, spikelets per panicle, seed setting rate and 1, 000-grain weight were also estimated at maturity stage for the parents, NIL36 and knockout lines.

All plants were planted in accordance with standard commercial practices. Plants were grown in the field at 13.3 cm plant to plant distance and 26.40 cm row to row distance. Agronomical practices were performed according to the normal agricultural practices locally recommended for rice production.

### Construction of a Mapping Population for Major QTLs Identification

The NIL36 line was crossed with the Jiafuzhan cultivar to develop a mapping population. The F_2_ population was constructed by self-crossing of the F_1_ hybrid. A primary linkage of the QTLs for plant height was obtained using 45 recessive plants from the F_2_ population. Furthermore, 1264 recessive plants from the F_2_ population were selected for fine mapping of major plant height QTLs.

### PCR Amplification and Marker Detection Analysis

The CTAB method (Murray and Thompson, 1980) with minor modifications was used for the extraction of plant DNA from frozen leaves of the rice plants. For PCR amplification, each 20-μl reaction mixture contained 30 ng DNA, 0.4 μm primers and 2× Es Tag MasterMix (Dye). The amplification program includes the following procedures: 2 min at 94°C; 33 cycles of 30 s at 94°C, 30 s at 55°C, and 30 s at 72°C; and a final extension at 72°C for 2 min. The amplified PCR products underwent 3% agarose gel electrophoresis and were stained with ethidium bromide (Panaud et al., 1996).

### Genetic Mapping of Plant Height QTLs

We used the obtained phenotypic data and SSR markers for the identification of QTLs. Genetic distance was estimated using MapDraw V2.1 (Liu and Meng, 2003). The genetic linkage map obtained in this study is basically consistent with that reported by Rahman et al. (2007).

### Physical Mapping and Bioinformatics Analysis of the Major Plant Height QTL *qph12*

The physical map of QTLs for plant height was constructed through a bioinformatics analysis using the published sequences of BAC and P1-derived artificial chromosome (PAC) clones of cv. Nipponbare released by the International Rice Genome Sequencing Project.^1^ Target gene linkage markers were used to clone, and sequence alignment was performed using the matching Basic Local Alignment Search Tool. According to the existing sequence annotation database,^2^ candidate genes based on the existing sequence annotation database analysis were identified.

### Targeted Knockout of Candidate Genes in Jiafuzhan Using the CRISPR/Cas9 Approach

The first exon of the *qPH12* gene in the Jiafuzhan cultivar was targeted with one gRNA spacer. Highly specific gRNA spacer sequences were designed using CRISPR plant database and website (gRNA: ggctgacgaccgggagaagaagg; Xie et al., 2014). The primer sequence for vector construction were showed (Supplementary Table 1). Genome editing mutations of target genes in regenerated plants were analyzed. The deletion and insertion within targeted genes were detected by PCR. PCR products were selected from transgenic CRISPR-edited strains for sequencing to identify specific mutations. The degradation sequence decoding method was used to analyze the double peaks (Ma et al., 2015). The primers used in CRISPR/Cas9 experiments are shown in Supplementary Table 1.

### Measuring the Levels of Phytohormones

Stems of Jiafuzhan (CK), NIL36, *qPH12KO-line1*, *qPH12KO-line2* and *qPH12KO-line3* were sampled during the rice jointing stage. The auxin (IAA) contents were measured using MetWare^3^ based on the AB Sciex QTRAP4500 LC–MS/MS platform. For the determination of auxin content, plants were sampled at the heading and jointing stage of rice with 3 replicates were sampled from each genotype. Samples were treated with methanol dissolved as a solvent and stored at −20°C. Samples were diluted into different gradient concentrations before mass spectrometry. The Ultra Performance Liquid Chromatography was used for separation. The Multiple Reaction Monitoring was implemented for the analysis.

### Expression Analysis of the *qPH12*

Total RNA was extracted from rice leaves according to the instructions of the extraction kit (TRIzol, Invitrogen, United States), and DNase treated. An aliquot of about 1.5 μg of RNA was reverse transcribed using the first strand cDNA synthesis kit (Bao Bioengineering Co., LTD.), and the cDNA was 10-times diluted for RT-qPCR. Primers for RT-qPCR were designed and optimized to >95% amplification efficiency. Fluorescence of the Real-Time Fluorescence Quantitative kit (Bao Bioengineering Co., Ltd.) was measured in an CFX96 real-time PCR apparatus (Bio-Rad, Munich, Germany) according to the RT-qPCR procedure described by Bustin et al. (2009). Expression levels were measured using three independent biological replicates and three technical replicates and normalized against the reference gene *UBIQUITIN*. Primer sequences of related genes are shown in Supplementary Table 1.

## Results

### Identification and Analysis of Major QTLs for Plant Height in the NILs

**Figure 1:**
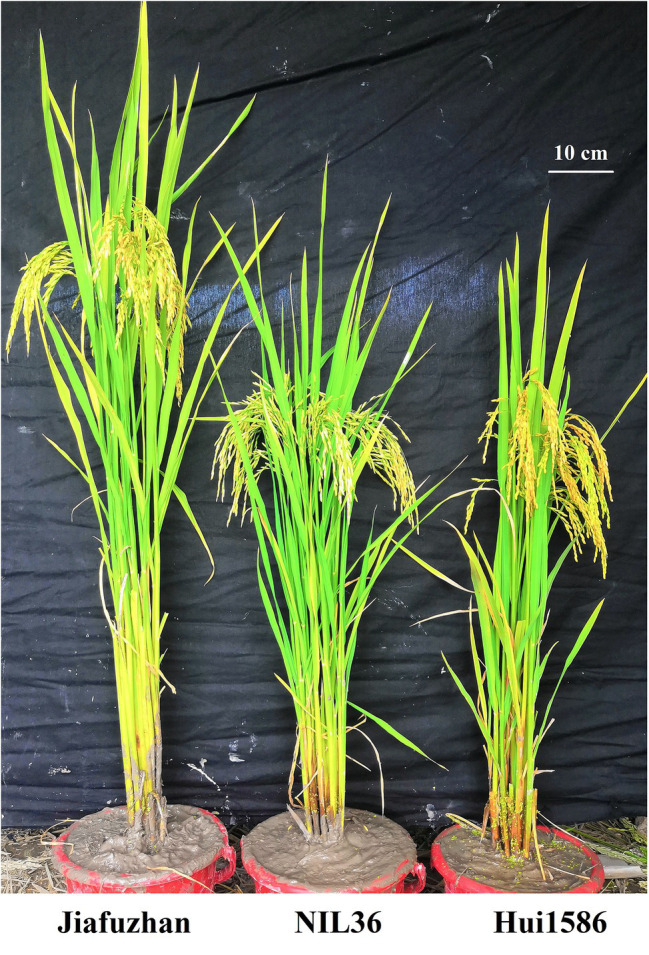
Phenotypic comparison of Jiafuzhan and NIL36. The phenotypes of Jiafuzhan and NIL36 at the mature period are shown.

To evaluate the potential advantages of the NILs for major QTLs detection, the phenotypic variations in plant height were observed in 176 NILs, with the NIL36 line exhibited a lower plant height compared to the Jiafuzhan cultivar, however, no significant differences were observed in plant height among the remaining NILs. QTL analysis using the WinQTLCart 2.5 software showed that the LOD score value of the plant height QTL for the NIL36 reached 7.84, with an explained phenotypic variance (*R*^2^) of 17.52%. Further investigations and analyses showed that the plant height of the Jiafuzhan plants was 116.22 cm, whereas that of the NIL36 line was 79.52 cm. The differences in plant height between these lines reached a highly significant level, as revealed by the *t*-test (Table 1; Figure 1).

**Table 1:** Comparison of the main agronomical traits of Hui1586, Jiafuzhan, NIL36 and the *qPH12KO* knockout mutant lines.

Traits	Hui1586	Jiafuzhan	NIL36	*qPH12KO-line1*	*qPH12KO-line2*	*qPH12KO-line3*
Plant height (cm)	85.62 ± 1.92^**^	116.22 ± 2.26	79.52 ± 1.72^**^	80.22 ± 1.76^**^	81.02 ± 1.82^**^	80.35 ± 1.81^**^
Panicle length (cm)	19.24 ± 1.18^*^	27.12 ± 1.12	21.26 ± 1.08^*^	22.36 ± 1.24^*^	22.66 ± 1.12^*^	21.96 ± 1.32^*^
Number of effective panicle	10.16 ± 1.18	10.54 ± 1.04	10.82 ± 1.08	10.42 ± 1.12	10.82 ± 1.18	10.72 ± 1.02
Spikelets per panicle	146.76 ± 3.98^*^	168.46 ± 4.86	125.86 ± 4.32^**^	128.76 ± 4.62^**^	130.12 ± 4.82^**^	122.86 ± 4.22^**^
Seed setting rate (%)	95.74 ± 1.46	97.52 ± 1.26	98.22 ± 1.18	97.28 ± 1.28	97.38 ± 1.18	96.98 ± 1.18
1,000-grain weight (g)	26.32 ± 0.64^*^	23.32 ± 0.54	23.44 ± 0.42	23.12 ± 0.48	23.54 ± 0.53	23.64 ± 0.46
Grain length (mm)	8.42 ± 0.21^*^	10.95 ± 0.13	10.72 ± 0.16	10.80 ± 0.20	10.92 ± 0.214	10.88 ± 0.15
Grain width (mm)	3.72 ± 0.09^**^	2.68 ± 0.08	2.72 ± 0.05	2.70 ± 0.08	2.66 ± 0.09	2.68 ± 0.07
Yield per plant (g)	37.57 ± 1.01^*^	40.38 ± 1.02	31.35 ± 0.98^**^	30.17 ± 1.08^**^	32.33 ± 1.02^**^	31.16 ± 1.08^**^

* Indicate the significance levels of the differences between Hui1586 and Jiafuzhan, NIL36 and knockout lines at *p* < 0.05, respectively. The data was derived from the trial that was performed at the Hainan experimental station in April 2019.

** Indicate the significance levels of the differences between Hui1586 and Jiafuzhan, NIL36 and knockout lines at *p* < 0.01, respectively. The data was derived from the trial that was performed at the Hainan experimental station in April 2019.

Phenotypic comparisons between the NIL36 and the Jiafuzhan plants are presented in Table 1. The results showed some significant differences in major agronomical traits, including plant height, panicle length, spikelets per panicle and yield per plant, between the NIL36 line and the Jiafuzhan cultivar. However, no significant differences observed in the number of effective panicles, seed setting rate, 1,000-grain weight, grain length or grain width (Table 1).

### Genetic Analysis of the Plant Height Phenotype in the NIL36

To determine whether plant height in the NIL36 line is controlled by a single gene or not, NIL36 was crossed to the Jiafuzhan cultivar. F_1_ plants showed the plant height phenotype of the Jiafuzhan cultivar, meanwhile the F_2_ population showed Mendelian segregation (Table 2). The segregation between the Jiafuzhan and NIL36 phenotypes fit the 3:1 segregation ratio in the two F_2_ populations (*χ*^2^ = 0.134 ~ 0.456, *p* > 0.5). The results showed that the plant height phenotype in the NIL36 is controlled by a single recessive gene.

**Table 2:** Phenotypic segregation for plant height in the F_2_ populations derived from crosses between the Jiafuzhan cultivar and the NIL36 lines.

Crosses	F_1_ phenotype	F_2_ population			*χ*^2^ test for H_0_ = 3:1	*P*
		Normal type of Jiafuzhan	Normal type of NIL36	Total Plants		
NIL36/Jiafuzhan	Normal type of Jiafuzhan	240	82	322	0.456^*^	0.5–0.75
Jiafuzhan/NIL36	Normal type of Jiafuzhan	286	90	376	0.134^*^	>0.9

* The segregation of the normal to mutated phenotype was 3:1 at the 0.05 significance level.

### Co-segregation Analysis of Plant Height Phenotype and the Major QTL in the NIL36

To identify the gene responsible for the NIL36 plant height phenotype, we located the plant height QTL in the NIL36 and a total of 506 SSR markers from the rice molecular map were selected for polymorphism surveys between Hui1586 and Jiafuzhan (Mccouch et al., 2002). Out of those 506 SSR markers, 296 exhibited polymorphisms between Hui1586 and Jiafuzhan cultivars. Based on the genotypic data of these 296 SSR markers, 45 recessive plants from the F_2_ population (NIL36/Jiafuzhan) were used for co-segregation analysis between the SSR markers and the plant height phenotype. One of these SSR markers, *RM3326*, that located on chromosome 12, showed a complete co-segregation with the plant height phenotype in the selected 45 F_2_ recessive individuals. The major QTL was therefore designated *qph12*.

Based on 296 polymorphisms exhibited between Hui1586 and Jiafuzhan cultivars, the genetic background of NIL36 homozygous material was analyzed using 145 SSR primer pairs distributed evenly on the 12 chromosomes of rice. The results showed that four markers, i.e., *RM6832* on chromosome 3, *RM3498* on chromosome 6, *RM3496* on chromosome 8, and *RM2584* on chromosome 12, exhibited the homozygous alleles of the Hui1586 cultivar., whereas the remaining 292 markers showed the genetic background of the Jiafuzhan cultivar (Supplementary Figure 2). Therefore, the NIL36 basically restores the genetic background of the recipient parent.

### Initial Localization of the *qph12* for Plant Height

**Figure 2:**
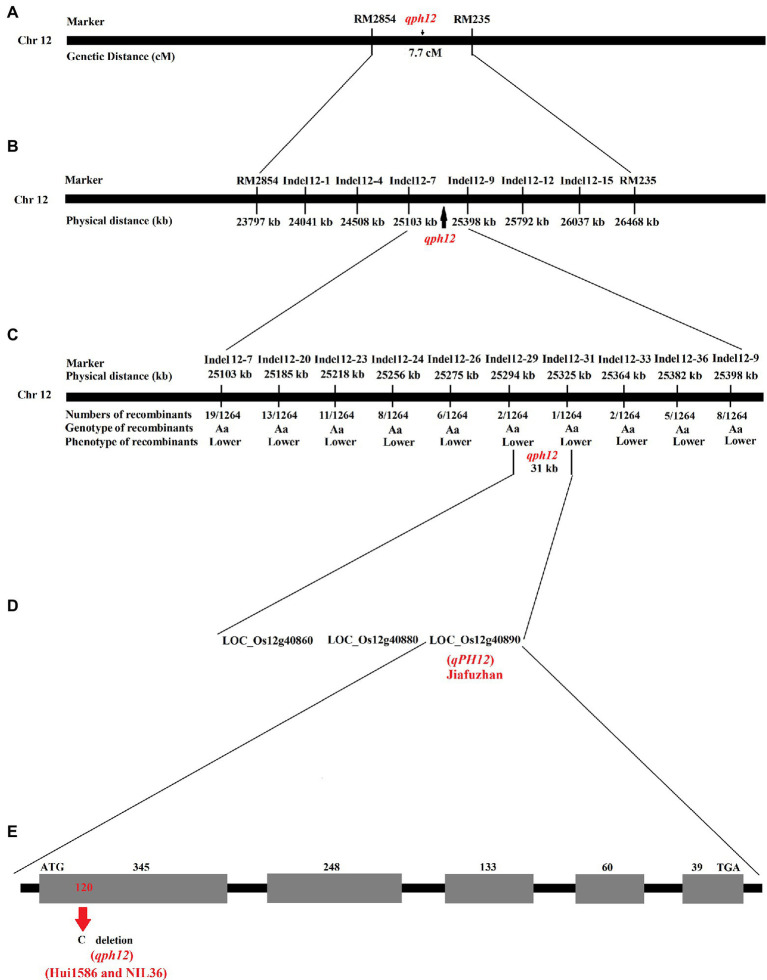
Physical maps and structural comparison of *qPH12*. **(A)** Primary mapping of *qPH12*. The gene was mapped to the region between the markers RM2854 and RM235. **(B)** Further mapping of *qPH12*. The gene was mapped to the region between markers Indel12-7 and Indel12-9. **(C)** Fine mapping of *qPH12*. *qPH12* was localized to a 31 kb region between the markers Indel12-29 and Indel12-31, and the recombinant number between the markers and target genes is indicated under the linkage map. **(D)** Candidate genes in the 31 kb target region. **(E)** *qPH12* has five exons, and *qph12* exhibits a 1 bp deletion in the first exon.

Publicly available molecular markers around *RM3326* marker were used to initially locate the *qph12* QTL. A genetic linkage analysis revealed that the *qph12* QTL is located between the molecular markers *RM2854* and *RM235*, which are located at a distance of 7.7 cM (Figure 2A). To delimit the genomic region of the *qph12*, 1264 recessive plants from the Jiafuzhan/NIL36 F_2_ population were genotyped using six polymorphic indel markers selected from 18 newly developed indel markers (Table 3). Indel markers from the open rice genome sequences were designed and tested to predict the likelihood of polymorphism between the NIL36 line and the Jiafuzhan cultivar by comparing sequences from *Nipponbare*^4^ and the *indica* cultivar 93–11.^5^ The genotyping of all recombinant genes was performed using six polymorphic markers. The results showed that the *qph12* QTL was located within a 295 kb region between the molecular markers *Indel12-7* and *Indel12-9* on chromosome 12 (Figure 2B; Table 3).

**Table 3:** Indel and SSR molecular markers used for fine mapping of the *qPH12*.

Marker	Sequence of the forward primer	Sequence of the reverse primer
RM3326	CTCATCACCATCGTCACCAC	TCGTCGGGAGAGAGAGAGAG
RM2854	ATGAGAGAGAGAAAGAGAGT	AATGGAGAGAAAAAGTATTA
RM235	AGAAGCTAGGGCTAACGAAC	TCACCTGGTCAGCCTCTTTC
Indel12-1	CACCATGGACGATTTCTCTTCG	GATCGATGAGCAAGAAGGAGAGC
Indel12-4	GCGAGGTGTTGTGGACGATGG	ACACCTCCATCTTGGCCTTCTCG
Indel12-7	ATCATCGTCGTCATCCTCTCTCC	CGTCCAGTTCGTAGGCGTATAAGG
Indel12-9	ACGGTGGTGGTGGTGTTGTCG	TTAACCTTTGGCCGGGAGTGTGG
Indel12-12	GGTGTTGATTAAGCTGATCTCTCTCC	GATCAGCAACAAGCACCTCAGC
Indel12-15	TTGCTACTACCACAACAGGGTTCC	GCAGCCACAGCTTTGAATAGAGC
Indel12-20	CAAACAGGGTGAAAGAGAGA	CCTTTGCTACCTTGTGCTAC
Indel12-23	TAGAAGAGTGGGACAAGGAA	TGTTCATTTACATGCACCAT
Indel12-24	ACATCGATCCATTGCTAGTT	ACATCACGTGGTGGTTTATT
Indel12-26	TTCAGATACCAACACCTCCT	TTTTCCCTGACATTGGATAC
Indel12-29	TGCTGAACTAATCTGTGTGC	ATCTTTTCCTTGGGTTTCAT
Indel12-31	CATACACACAACAAATAGAA	CGCCAATCTTTAAATAGTTT
Indel12-33	ACACGTCTTTTCTGCAAGAT	GAACGAACATGAACGAGCTA
Indel12-36	TGGATGCATGGTAACTAATG	TGAATTGCTCTCCATGAAAT

### Fine Mapping of the Plant Height QTL *qph12*

For fine mapping of the *qph12* QTL, eight polymorphic indel markers were selected from 26 newly developed indel markers (Table 3). Recombinant screening with eight markers located in a more internal position within the target locus detected 13, 11, eight, six, two, one, two, and five recombinant plants, respectively (Figure 2C). Thus, the *qph12* locus was precisely located within a 31 kb region between the molecular markers *Indel12-29* and *Indel12-31*.

### Candidate Genes Identification Within the 31 Kb Region of the *qph12* Locus

According to the publicly available sequence annotation databases,^6^^,^^7^ three annotated genes with a corresponding full-length cDNA are located within the 31 kb region (Figure 2D). Among these genes, *LOC_Os12g40860* encodes the leucine-rich repeat family protein, *LOC_Os12g40880* encodes the uridine kinase family protein, and *LOC_Os12g40890* is the auxin-responsive *Aux/IAA* gene family member *OsIAA30*.

### Sequence Analyses of the Plant Height QTL *qph12*

To identify the gene responsible for the observed plant height phenotype, we sequenced the three candidate genes in the Jiafuzhan cultivar and the NIL36 line. A deletion of only 1 bp (120:C) was found in the *LOC_Os12g40890* gene in the NIL36 (Figure 2E), and no further difference was observed between the sequences of the remaining two genes in the two genotypes. Thus, we hypothesized that the recessive gene corresponds to the major plant height QTL *qph12* in the NIL36 line, and the dominant allele (*LOC_Os12g40890*) of the WT Jiafuzhan was designated *qPH12*. Since the fragment of *qph12* was probably derived from the parent Hui1586, we sequenced the *qph12* of Hui1586, and the results showed that the sequences of *qph12* from both Hui1586 and NIL36 are identical (Figure 2E).

The analysis of the open reading frame (ORF) region showed that the *qPH12* gene has five exons. *qph12* exhibited a 1 bp deletion in the 120th bp of the first exon, which resulted in premature termination of the *qPH12* (Figure 2E).

### The *qph12* Is Responsible for the Plant Height Phenotype in the NIL36

**Figure 3:**
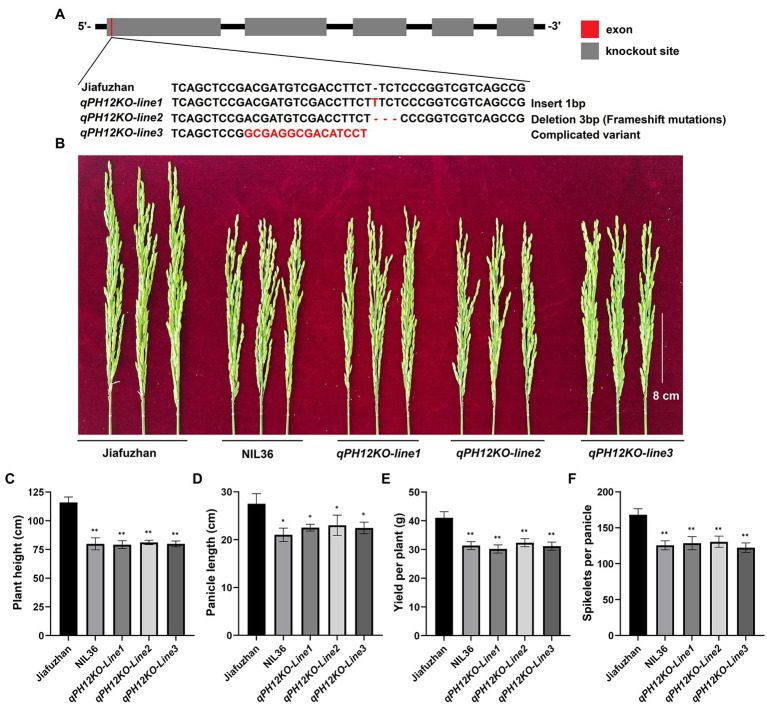
*qPH12-knockout lines* generated using CRISPR/Cas9 technology and showed the NIL36 phenotype. **(A)** Three independent events (designated *qPH12KO-line1*, *qPH12KO-line2* and *qPH12KO-line3*) were generated using the CRISPR/Cas9 system and verified by sequencing. **(B)** Panicle differences of Jiafuzhan, NIL36 and knockout lines; **(C–F)** indicate the differences of plant height, panicle length, spikelets per panicle and yield per plant, respectively, across Jiafuzhan, NIL36 and knockout lines in Table 1. ^*^Statistical significance (*p* < 0.05) determined using Student’s *t*-test. ^*^^*^Statistical significance (*p* < 0.01) determined using Student’s *t*-test.

To confirm that *qph12* confers the plant height phenotype, we examined whether the knockout of *qPH12* in the Jiafuzhan cultivar would lead to the NIL36 phenotype. One sequence-specific guide RNA (sgRNA) was designed to knock out *qPH12* using the CRISPR/Cas9 gene editing system. After resistance screening, 20 strains were randomly selected for sequencing. The results revealed four main types of mutations, i.e., insertion, deletion, complex variant and no mutation, from which insertion and deletion T_1_ homozygous genotypes were self-pollinated for two generations to produce the T_3_ generations, designated as *qPH12KO-line1* and *qPH12KO-line2*, respectively. The complicated variant T_1_ replaced the heterozygous genotype, and the T_2_ generation was sequenced and screened, and self-pollinated for three generations to produce the T_4_ generation, *qPH12KO-line3*. A total of three plants from three independent events (*qPH12KO-line1*, *qPH12KO-line2* and *qPH12KO-line3*) were obtained. Sequencing of the *qPH12* gene in the three independent knockout lines confirmed that these plants carry mostly insertion or deletion in the targeted sites (Figure 3A). Evaluation of the plant height phenotype of these three homozygous lines at maturity and found that all three lines showed the NIL36 phenotype (Table 1; Figure 3B). Therefore, the targeted mutation of the *qPH12* gene led to the NIL36 plant height phenotype, indicating that the loss of function of *qPH12* was responsible for the reduced plant height phenotype. Notably, the three knockout lines *qPH12KO-line1*, *qPH12KO-line2* and *qPH12KO-line3* showed shorter panicle length, fewer number spikelets per panicle and lower plants yields compared to the Jiafuzhan cultivar (Table 1; Figures 3C–F). Therefore, we hypothesized that the *qph12* QTL not only affects plant height but also underlies panicle length, spikelets per panicle and plant yield in rice. According to the standard commercial practices, *qPH12KO-line1*, *qPH12KO-line2*, *qPH12KO-line3* and Jiafuzhan were grown in a paddy field under natural environmental conditions at transgenic experimental base in Fuzhou, China.

### Comparative Analysis of the Phytohormones Content Between the Jiafuzhan Cultivar and the Three *qPH12KO* Knockout Lines

**Figure 4:**
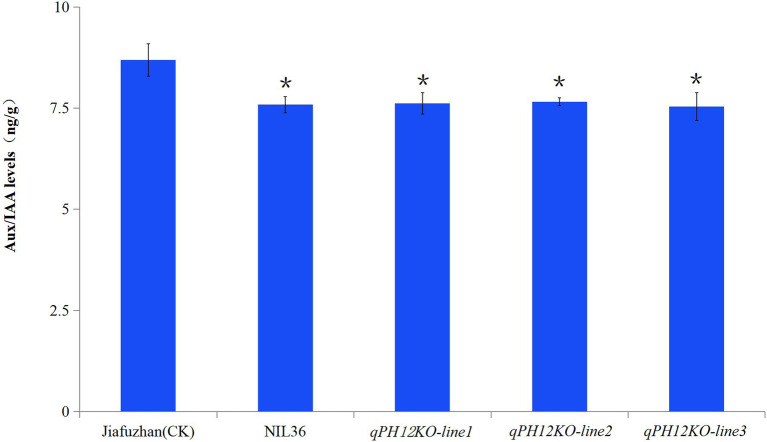
Comparison of Aux/IAA levels between Jiafuzhan and *qPH12KO-lines*. The Aux/IAA content of *qPH12KO-line1, qPH12KO-line2, qPH12KO-line3* and NIL36 was significantly lower than that of the Jiafuzhan cultivar (CK). Three experimental replicates of each line were included. ^*^Statistical significance (*p* < 0.05) determined using Student’s *t*-test.

To analyze whether the *qph12* QTL affects the changes in the Aux/IAA levels, we measured the Aux/IAA content in the Jiafuzhan (CK) and the *qPH12KO* knockout lines. The results showed that the Aux/IAA content in *qPH12KO-line1*, *qPH12KO-line2*, *qPH12KO-line3* and NIL36 line was significantly lower than that of the Jiafuzhan (CK; Figure 4).

### Jiafuzhan Accumulates More *qPH12* Transcript

**Figure 5:**
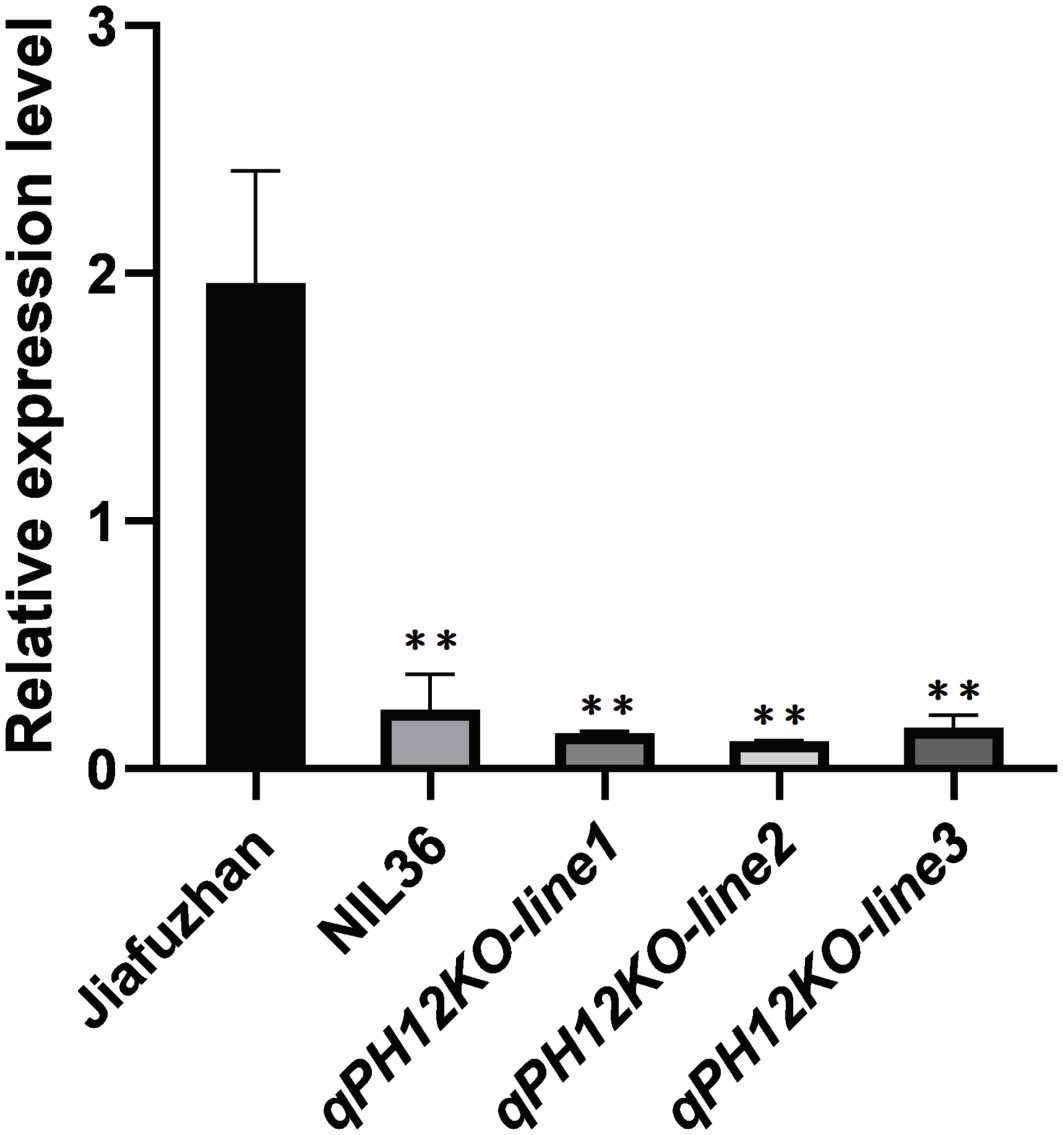
RT-qPCR expression analysis of *LOC_Os12g40890* in Jiafuzhan, NIL36 and the three *qPH12KO* knockout mutant lines. The y-axis represents the relative expression value (log_2_ – transformed, mean ± SD, *n* = 3 biological replicates). ^**^significance differences (*p* < 0.01) determined by the Student’s *t*-test; ns, non-significant difference.

To further investigate the expression abundance of *qPH12* gene in Jiafuzhan, NIL36 and the three *qPH12KO* knockout mutant lines, we analyzed the expression profile of *qPH12* in these materials using RT-qPCR. The *qPH12* expression was significantly higher in the Jiafuzhan that showed higher plant height and grain yield, longer panicles and more spikelets per panicle compared to the NIL36 line and the three knockout mutant lines *qPH12KO-line1*, *qPH12KO-line2* and *qPH12KO-line3* that showed a reduced plant height phenotype (Figure 5).

## Discussion

NIL populations have been developed and used for genetic studies and fine mapping of QTLs for genome-wide target traits (Yang et al., 2016). Each NIL carries one or more donor chromosome segments, which provide distinct advantages for QTLs identification, and a QTL can be visualized as a single Mendelian factor by blocking background genetic noise. Different types of QTLs have been identified and/or cloned using NIL populations, such as the drought resistance, *qDTY 2.2* (Henry et al., 2015); the thermotolerance, *TT1* (Li et al., 2015); the cold tolerance, QTLs (Zhou et al., 2012), the grain type, *GS3* (Fan et al., 2006) and *GW2* (Song et al., 2007); the heading stage, *Ghd7* (Xue et al., 2008) and *qHD19* (Yang et al., 2020); the spike type, *DEP1* (Huang et al., 2009); the grain production, *Cn1a* (Ashikari et al., 2005) and 99 QTLs for different agronomic traits (Furuta et al., 2014).

Although a number of plant height related genes/QTLs have been identified, and these genes/QTLs tend to have a function in hormone biosynthesis pathways including gibberellic acid, brassinosteroid, and strigolactone (Shearman et al., 2019), there are also hundreds of QTLs where the underlying cause remains unknown (Lei et al., 2018). In the present study, we developed 176 NILs with a genetic background from the *indica* rice cultivar Jiafuzhan. Using these lines, we mapped a major plant height QTL designated as *qph12*, which encoded the auxin-responsive family protein Aux/IAA. Further analysis revealed that *qph12* is a novel plant height QTL and its molecular function has not yet been identified. Map-based cloning and knockout experiments confirmed that the reduced plant height phenotype of the NIL36 line is caused by the loss of function of *qPH12* that residing the major plant height QTL *qph12*. Further analyses revealed that the reduced plant height phenotype resulting from the *qph12* QTL is caused by a functional deletion in the *qPH12* gene. The sequencing results showed that the main cause of this mutation could be due to the mutation in the Hui1586 parent. Similarly, Shearman et al. (2019) reported that the reduced plant height phenotype resulted from the introgression segments from IR62266 into KDML 105 is due to that the IR62266 has a deletion in the G*ibberellin 20-oxidase 2* gene that corresponds to the *semi-dwarf 1* locus, which led to the identification of a plant height QTL.

Previous studies have showed that *Aux/IAA* genes play important roles in plant growth and development by regulating the expression of early auxin-responsive genes (Jain et al., 2006). For example, *OsIAA1* and *OsIAA3* affect plant morphogenesis (Thakur et al., 2001; Nakamura et al., 2006), *OsIAA11* and *OsIAA13* affect root development in rice (Kitomi et al., 2012; Zhu et al., 2012), and *OsIAA6* is involved in tiller outgrowth (Jung et al., 2015). The present study showed that *qph12* exhibits a 1 bp deletion in the first exon of *qPH12* which is the auxin-responsive *Aux/IAA* gene family member *OsIAA30.* Further research showed that the *qph12* QTL significantly reduces plant height in rice. Besides, the knockout experiments confirmed that *qph12* QTL is responsible for the plant height phenotype. Three homozygous knockout lines also exhibited the phenotype of the NIL36 line, which includes a reduced plant height, a shorter panicle length, fewer spikelets per panicle and a lower yield per plant compared to the Jiafuzhan cultivar (Table 1). Analysis of the phytohormone content showed that the Aux/IAA contents in the NIL36 line, *qPH12KO-line1*, *qPH12KO-line2, qPH12KO-line3* were significantly lower than that of the Jiafuzhan (CK) cultivar (Figure 4). These findings suggest that *qph12* QTL simultaneously regulates plant height, panicle length, spikelets per panicle and yield per plant by manipulating the auxin levels in the plants.

In addition, the expression level of *qPH12* was higher than that of NIL36 and the three knockout mutant lines (Figure 5). We speculated that this might be a feedback regulation. Under favorable conditions, the downstream signal would promote the expression of *qPH12* in response to the activation of *qPH12* signaling pathway. Meanwhile, the *qPH12* mutant (*qph12*) would not activate the downstream signaling pathway, so the downstream components could not perceive this biological signal and the expression of *qPH12* would no longer needed, and therefore the *qPH12* expression level is reduced.

Although the *qph12* and the *qPH12* affects certain traits, such as plant height, panicle length, spikelets per panicle and yield per plant, the question should be answered, whether *qph12* and *qPH12* alleles have specific application prospects in the improvement of rice breeding? To address this question, we performed SNP (Single nucleotide polymorphisms) calling and haplotype analysis of the 3,000 sequenced rice genomes available in the CNCGB and CAAS databases (Li et al., 2014) and found 65 haplotypes for the *qPH12* gene, including 6 haplotypes among more than 15 rice resource materials (Supplementary Table 2). Further analysis revealed that the Hap1 of the *qPH12* contained 2453 rice resource materials, which showed that the *qPH12* was predominantly existed in rice population. However, fewer haplotypes were found for the *qph12* in the 3,000 sequenced rice genomes. Therefore, to breed a new hybrid rice variety with an ideal plant height, breeders can transfer *qph12* into both restorer and sterile lines through molecular marker-assisted selection.

## Data Availability Statement

The original contributions presented in the study are included in the article/Supplementary Material, further inquiries can be directed to the corresponding author.

## Author Contributions

DY planned and performed the experiments and data collection and wrote the manuscript with input from all authors. NH, GZ, and FH were involved in conducting the experiment, data collection, and analyses. SA-E revised the manuscript. All authors discussed the results and contributed to the final manuscript.

## Funding

The work was supported by the Special Fund for Agro-scientific Research in the Public Interest of Fujian Province (no. 2020R1023003), the Fujian Provincial Natural Science Foundation of China (no. 2021J01471), Major Science and Technology Projects of Fujian Province (no. 2020NZ08016), Science and Technology Innovation Team (no. CXTD2021001), 5511 Collaborative Engineering Project (no. KXXYJBG0021), and the 100 Talent Plans of Fujian Province.

## Conflict of Interest

The authors declare that the research was conducted in the absence of any commercial or financial relationships that could be construed as a potential conflict of interest.

## Publisher’s Note

All claims expressed in this article are solely those of the authors and do not necessarily represent those of their affiliated organizations, or those of the publisher, the editors and the reviewers. Any product that may be evaluated in this article, or claim that may be made by its manufacturer, is not guaranteed or endorsed by the publisher.

## Supplementary Material

The Supplementary Material for this article can be found online at: https://www.frontiersin.org/articles/10.3389/fpls.2022.878558/full#supplementary-material

## Footnotes

^1^ http://rgp.dna.affrc.go.jp/IRGSP/index.html

^2^http://rice.plantbiology.msu.edu/; http://www.tigr.org/

^3^ http://www.metware.cn/

^4^ http://rgp.dna.affrc.go.jp/

^5^ http://rice.genomics.org.cn/

^6^ http://rice.plantbiology.msu.edu/

^7^ http://www.tigr.org/
