# Supplementary material for: Fine Mapping and Cloning of a Major QTL qph12, Which Simultaneously Affects the Plant Height, Panicle Length, Spikelet Number and Yield in Rice (Oryza sativa L.)
Source: Front Plant Sci. 2022 May 27;13:878558. doi: 10.3389/fpls.2022.878558 (PMC9187155; doi:10.3389/fpls.2022.878558)
Supplement: Supplementary file 1 [file Data_Sheet_1.docx]

**Supplementary Table 1.** Sequences of primers used in the study.

| Name | Sequence (5’→3’) | Purpose |
| --- | --- | --- |
| gRNAs-*LOC_Os12g40890* | ggctgacgaccgggagaagaagg | CRISPR/Cas9 |
| *LOC_Os12g40890-*F1 | CCTTCGAGGCGACCGAGCTC | Screening of lines |
| *LOC_Os12g40890-*R1 | CTGTGCACGTACTTGGGAGC | Screening of lines |
| *LOC_Os12g40890-*qRT-PCR-F | CCTTCGTGAAGGTGAGCATGG | RT-qPCR |
| *LOC_Os12g40890-*qRT-PCR-R | GCCGTTGAGCAGATCAGCAAT | RT-qPCR |
| *UBIQUITIN*-F | AACCAGCTGAGGCCCAAGA | Reference gene |
| *UBIQUITIN*-R | ACGATTGATTTAACCAGTCCATGA | Reference gene |

**Supplementary Table 2.** Haplotype analysis of *qPH12*

| Haplotype | SNP | Sample list | Group | Plant Height (2666) |
| --- | --- | --- | --- | --- |
| Hap1 | ATGCG | [2453](https://www.rmbreeding.cn/downloads/haplotype/2yZsM46sCK3906311.txt) | Aus: 65Bas: 38; GJ: 794; XI: 1477; admix: 78; na: 1 | (2170 of 2453) mean: 113.643 |
| Hap2 | GCCAG | [138](https://www.rmbreeding.cn/downloads/haplotype/2yZsM46sCK3906312.txt) | XI: 138 | (114 of 138) mean: 124.462 |
| Hap3 | GTCAC | [133](https://www.rmbreeding.cn/downloads/haplotype/2yZsM46sCK3906313.txt) | Aus: 108; Bas: 15; XI: 6; admix: 4 | (117 of 133) mean: 112.261 |
| Hap4 | AT-CG | [74](https://www.rmbreeding.cn/downloads/haplotype/2yZsM46sCK3906314.txt) | Bas: 2; GJ: 22; XI: 48; admix: 2 | (67 of 74) mean: 110.952 |
| Hap5 | ATG-G | [54](https://www.rmbreeding.cn/downloads/haplotype/2yZsM46sCK3906315.txt) | Aus: 2; Bas: 3; GJ: 9; XI: 37; admix: 3 | (50 of 54) mean: 110.444 |
| Hap6 | RTGCG | [27](https://www.rmbreeding.cn/downloads/haplotype/2yZsM46sCK3906316.txt) | Aus: 1; Bas: 13; GJ: 7; XI: 5; admix: 1 | (22 of 27) mean: 104.964 |
| Hap7 | ATGC- | [13](https://www.rmbreeding.cn/downloads/haplotype/2yZsM46sCK3906317.txt) | GJ: 3; XI: 9; admix: 1 | (11 of 13) mean: 113.582 |
| Hap8 | GC-AG | [10](https://www.rmbreeding.cn/downloads/haplotype/2yZsM46sCK3906318.txt) | XI: 10 | (9 of 10) mean: 128.389 |
| Hap9 | AT--G | [10](https://www.rmbreeding.cn/downloads/haplotype/2yZsM46sCK3906319.txt) | GJ: 1; XI: 8; admix: 1 | (9 of 10) mean: 111.667 |
| Hap10 | RTSMS | [9](https://www.rmbreeding.cn/downloads/haplotype/2yZsM46sCK39063110.txt) | Aus: 2; Bas: 1; XI: 2; admix: 4 | (8 of 9) mean: 115.725 |
| Hap11 | RYSMG | [8](https://www.rmbreeding.cn/downloads/haplotype/2yZsM46sCK39063111.txt) | XI: 8 | (6 of 8) mean: 130.800 |
| Hap12 | GT-AC | [5](https://www.rmbreeding.cn/downloads/haplotype/2yZsM46sCK39063112.txt) | Aus: 5 | (4 of 5) mean: 104.525 |
| Hap13 | ATSCG | [5](https://www.rmbreeding.cn/downloads/haplotype/2yZsM46sCK39063113.txt) | GJ: 2; XI: 3 | (5 of 5) mean: 110.420 |
| Hap14 | GTGCG | [4](https://www.rmbreeding.cn/downloads/haplotype/2yZsM46sCK39063114.txt) | Aus: 3; GJ: 1 | (2 of 4) mean: 132.650 |
| Hap15 | A-GCG | [4](https://www.rmbreeding.cn/downloads/haplotype/2yZsM46sCK39063115.txt) | Bas: 1; GJ: 1; XI: 1; admix: 1 | (4 of 4) mean: 113.900 |
| Hap16 | ATG-- | [4](https://www.rmbreeding.cn/downloads/haplotype/2yZsM46sCK39063116.txt) | GJ: 1; XI: 2; admix: 1 | (3 of 4) mean: 108.800 |
| Hap17 | ----- | [3](https://www.rmbreeding.cn/downloads/haplotype/2yZsM46sCK39063117.txt) | Aus: 1; GJ: 1; XI: 1 | (3 of 3) mean: 105.300 |
| Hap18 | AT-C- | [3](https://www.rmbreeding.cn/downloads/haplotype/2yZsM46sCK39063118.txt) | XI: 3 | (3 of 3) mean: 122.500 |
| Hap19 | GCC-G | [3](https://www.rmbreeding.cn/downloads/haplotype/2yZsM46sCK39063119.txt) | XI: 3 | (3 of 3) mean: 128.767 |
| Hap20 | GTSAC | [3](https://www.rmbreeding.cn/downloads/haplotype/2yZsM46sCK39063120.txt) | Aus: 2; Bas: 1 | (2 of 3) mean: 91.200 |
| Hap21 | ATGMG | [3](https://www.rmbreeding.cn/downloads/haplotype/2yZsM46sCK39063121.txt) | Aus: 1; XI: 2 | (2 of 3) mean: 104.500 |
| Hap22 | ATGAG | [3](https://www.rmbreeding.cn/downloads/haplotype/2yZsM46sCK39063122.txt) | XI: 3 | (3 of 3) mean: 108.567 |
| Hap23 | A-G-G | [3](https://www.rmbreeding.cn/downloads/haplotype/2yZsM46sCK39063123.txt) | GJ: 2; XI: 1 | (3 of 3) mean: 126.033 |
| Hap24 | -TGCG | [2](https://www.rmbreeding.cn/downloads/haplotype/2yZsM46sCK39063124.txt) | GJ: 1; XI: 1 | (2 of 2) mean: 118.250 |
| Hap25 | ATGCS | [2](https://www.rmbreeding.cn/downloads/haplotype/2yZsM46sCK39063125.txt) | GJ: 2 | (2 of 2) mean: 96.550 |
| Hap26 | ATCCG | [2](https://www.rmbreeding.cn/downloads/haplotype/2yZsM46sCK39063126.txt) | GJ: 2 | (2 of 2) mean: 117.050 |
| Hap27 | GTCAS | [2](https://www.rmbreeding.cn/downloads/haplotype/2yZsM46sCK39063127.txt) | Aus: 2 | (2 of 2) mean: 104.200 |
| Hap28 | GCCA- | [2](https://www.rmbreeding.cn/downloads/haplotype/2yZsM46sCK39063128.txt) | XI: 2 | (1 of 2) mean: 115.900 |
| Hap29 | RYGCG | [2](https://www.rmbreeding.cn/downloads/haplotype/2yZsM46sCK39063129.txt) | GJ: 1; XI: 1 | (2 of 2) mean: 128.550 |
| Hap30 | AT--- | [2](https://www.rmbreeding.cn/downloads/haplotype/2yZsM46sCK39063130.txt) | Bas: 1; XI: 1 | (2 of 2) mean: 119.950 |
| Hap31 | GTC-C | [2](https://www.rmbreeding.cn/downloads/haplotype/2yZsM46sCK39063131.txt) | Aus: 2 | (2 of 2) mean: 114.650 |
| Hap32 | RTSCG | [2](https://www.rmbreeding.cn/downloads/haplotype/2yZsM46sCK39063132.txt) | XI: 1; admix: 1 | (0 of 2) mean: NA |
| Hap33 | GTCMC | [2](https://www.rmbreeding.cn/downloads/haplotype/2yZsM46sCK39063133.txt) | Aus: 2 | (2 of 2) mean: 99.100 |
| Hap34 | RTSAS | [1](https://www.rmbreeding.cn/downloads/haplotype/2yZsM46sCK39063134.txt) | admix: 1 | (1 of 1) mean: 105.100 |
| Hap35 | GTCCG | [1](https://www.rmbreeding.cn/downloads/haplotype/2yZsM46sCK39063135.txt) | admix: 1 | (1 of 1) mean: 69.100 |
| Hap36 | ATGAC | [1](https://www.rmbreeding.cn/downloads/haplotype/2yZsM46sCK39063136.txt) | Aus: 1 | (1 of 1) mean: 115.800 |
| Hap37 | AYGCG | [1](https://www.rmbreeding.cn/downloads/haplotype/2yZsM46sCK39063137.txt) | GJ: 1 | (1 of 1) mean: 139.100 |
| Hap38 | -TCAC | [1](https://www.rmbreeding.cn/downloads/haplotype/2yZsM46sCK39063138.txt) | Aus: 1 | (1 of 1) mean: 99.800 |
| Hap39 | RTGC- | [1](https://www.rmbreeding.cn/downloads/haplotype/2yZsM46sCK39063139.txt) | XI: 1 | (1 of 1) mean: 111.400 |
| Hap40 | GCSAG | [1](https://www.rmbreeding.cn/downloads/haplotype/2yZsM46sCK39063140.txt) | XI: 1 | (1 of 1) mean: 147.800 |
| Hap41 | RYGAG | [1](https://www.rmbreeding.cn/downloads/haplotype/2yZsM46sCK39063141.txt) | XI: 1 | (1 of 1) mean: 130.000 |
| Hap42 | G-CAC | [1](https://www.rmbreeding.cn/downloads/haplotype/2yZsM46sCK39063142.txt) | Aus: 1 | (1 of 1) mean: 119.000 |
| Hap43 | --GC- | [1](https://www.rmbreeding.cn/downloads/haplotype/2yZsM46sCK39063143.txt) | GJ: 1 | (1 of 1) mean: 106.300 |
| Hap44 | A---G | [1](https://www.rmbreeding.cn/downloads/haplotype/2yZsM46sCK39063144.txt) | GJ: 1 | (1 of 1) mean: 146.200 |
| Hap45 | RYCMG | [1](https://www.rmbreeding.cn/downloads/haplotype/2yZsM46sCK39063145.txt) | XI: 1 | (1 of 1) mean: 114.600 |
| Hap46 | ATS-G | [1](https://www.rmbreeding.cn/downloads/haplotype/2yZsM46sCK39063146.txt) | Aus: 1 | (0 of 1) mean: NA |
| Hap47 | RT-CG | [1](https://www.rmbreeding.cn/downloads/haplotype/2yZsM46sCK39063147.txt) | admix: 1 | (0 of 1) mean: NA |
| Hap48 | RT-MS | [1](https://www.rmbreeding.cn/downloads/haplotype/2yZsM46sCK39063148.txt) | admix: 1 | (1 of 1) mean: 130.400 |
| Hap49 | ATGMS | [1](https://www.rmbreeding.cn/downloads/haplotype/2yZsM46sCK39063149.txt) | XI: 1 | (1 of 1) mean: 125.800 |
| Hap50 | RY-M- | [1](https://www.rmbreeding.cn/downloads/haplotype/2yZsM46sCK39063150.txt) | XI: 1 | (1 of 1) mean: 119.100 |
| Hap51 | GTCAG | [1](https://www.rmbreeding.cn/downloads/haplotype/2yZsM46sCK39063151.txt) | XI: 1 | (1 of 1) mean: 156.100 |
| Hap52 | GTCC- | [1](https://www.rmbreeding.cn/downloads/haplotype/2yZsM46sCK39063152.txt) | admix: 1 | (1 of 1) mean: 92.100 |
| Hap53 | ATSMS | [1](https://www.rmbreeding.cn/downloads/haplotype/2yZsM46sCK39063153.txt) | GJ: 1 | (1 of 1) mean: 115.100 |
| Hap54 | RYGMG | [1](https://www.rmbreeding.cn/downloads/haplotype/2yZsM46sCK39063154.txt) | XI: 1 | (1 of 1) mean: 135.300 |
| Hap55 | GYCAG | [1](https://www.rmbreeding.cn/downloads/haplotype/2yZsM46sCK39063155.txt) | XI: 1 | (1 of 1) mean: 125.100 |
| Hap56 | GTCA- | [1](https://www.rmbreeding.cn/downloads/haplotype/2yZsM46sCK39063156.txt) | XI: 1 | (1 of 1) mean: 137.400 |
| Hap57 | RYSAG | [1](https://www.rmbreeding.cn/downloads/haplotype/2yZsM46sCK39063157.txt) | XI: 1 | (0 of 1) mean: NA |
| Hap58 | GC--G | [1](https://www.rmbreeding.cn/downloads/haplotype/2yZsM46sCK39063158.txt) | XI: 1 | (1 of 1) mean: 112.300 |
| Hap59 | --GCG | [1](https://www.rmbreeding.cn/downloads/haplotype/2yZsM46sCK39063159.txt) | XI: 1 | (1 of 1) mean: 117.100 |
| Hap60 | RY-MS | [1](https://www.rmbreeding.cn/downloads/haplotype/2yZsM46sCK39063160.txt) | admix: 1 | (1 of 1) mean: 116.200 |
| Hap61 | ATSMG | [1](https://www.rmbreeding.cn/downloads/haplotype/2yZsM46sCK39063161.txt) | XI: 1 | (1 of 1) mean: 115.000 |
| Hap62 | RT--G | [1](https://www.rmbreeding.cn/downloads/haplotype/2yZsM46sCK39063162.txt) | Bas: 1 | (1 of 1) mean: 114.900 |
| Hap63 | RYSCG | [1](https://www.rmbreeding.cn/downloads/haplotype/2yZsM46sCK39063163.txt) | XI: 1 | (1 of 1) mean: 136.400 |
| Hap64 | GCS-G | [1](https://www.rmbreeding.cn/downloads/haplotype/2yZsM46sCK39063164.txt) | XI: 1 | (1 of 1) mean: 90.500 |
| Hap65 | GTSAS | [1](https://www.rmbreeding.cn/downloads/haplotype/2yZsM46sCK39063165.txt) | Aus: 1 | (1 of 1) mean: 132.800 |
