# Supplementary figures and images for: Fine Mapping and Cloning of a Major QTL qph12, Which Simultaneously Affects the Plant Height, Panicle Length, Spikelet Number and Yield in Rice (Oryza sativa L.)
Source: Front Plant Sci. 2022 May 27;13:878558. doi: 10.3389/fpls.2022.878558 (PMC9187155; doi:10.3389/fpls.2022.878558)

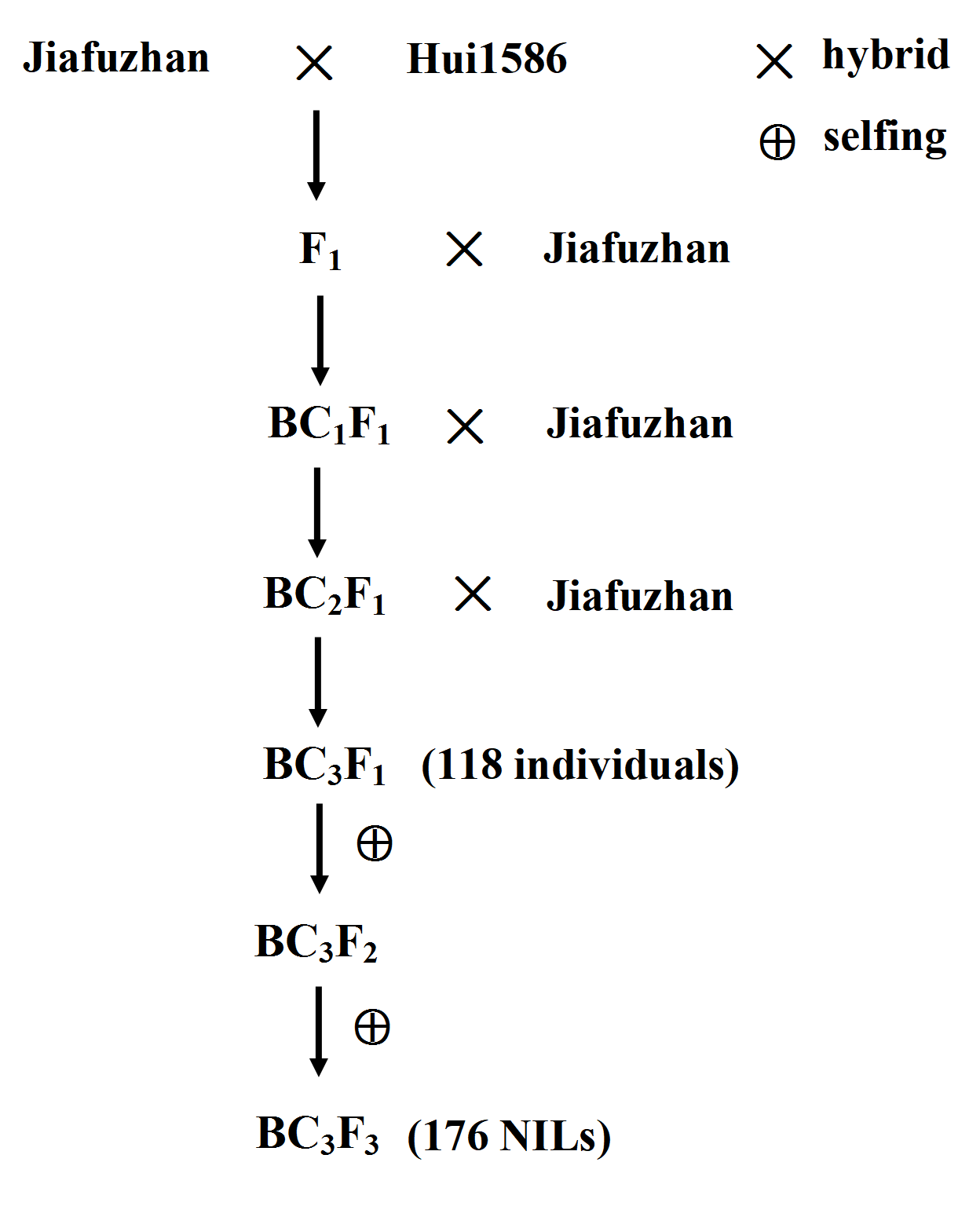

Supplement: Supplementary file 2 [file Image_1.TIF]

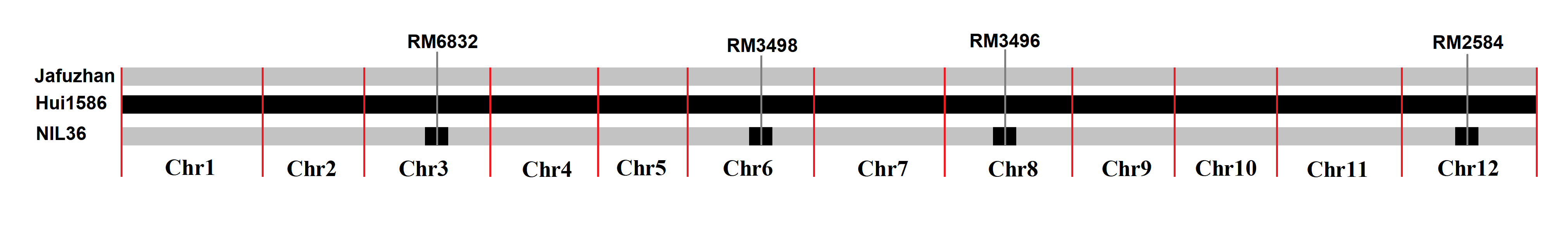

Supplement: Supplementary file 3 [file Image_2.PNG]
